# Protein expression dynamics during Escherichia Coli glucose-lactose diauxie

**DOI:** 10.1186/1471-2180-11-126

**Published:** 2011-06-01

**Authors:** Ekaterina Mostovenko, André M Deelder, Magnus Palmblad

**Affiliations:** 1 Department of Parasitology, Biomolecular Mass Spectrometry Unit, Leiden University Medical Center, P.O. Box 9600, Zone L04-Q, 2300 RC Leiden, Netherlands

**Keywords:** label-free, quantitative mass spectrometry, β-galactosidase, *Escherichia coli*, glucose-lactose diauxie

## Abstract

**Background:**

*Escherichia coli *is a well-studied anaerobic bacteria which is able to regulate metabolic pathways depending on the type of sugar presented in the medium. We have studied the glucose-lactose shift in *E. coli *at the protein level using a recently developed mass spectrometry platform.

**Method:**

Cells were grown in minimal medium containing two sugars (glucose and lactose) and analyzed using novel mass spectrometry cluster. The cluster combines the high resolving power and dynamic range of Fourier transform ion cyclotron resonance (FTICR) for accurate mass measurement and quantitation with multiple ion traps for fast and sensitive tandem mass spectrometry. The protein expression profile was followed in time across the glucose-lactose diauxic shift using label-free quantitation from the FTICR data.

**Results and Conclusion:**

The entire dataset was interrogated by KEGG pathway analysis, mapping measured changes in protein abundance onto known metabolic pathways. The obtained results were consistent with previously published gene expression data, with β-galactosidase being the most strongly induced protein during the diauxic shift.

## Background

Bacteria, such as *Escherichia coli*, provide "simple" biological models due to a relatively small genome/proteome size (less than 5,000 genes/proteins) and are easy to culture. When the growth medium is rich in glucose, *E. coli *uses glycolysis to convert glucose into pyruvate, requiring adenosine diphosphate (ADP) and oxidized nicotinamide adenine dinucleotide (NAD^+^) as cofactors. But *E. coli *is also able to use many other sugars, including lactose, as the main carbon source [1]. The genetic mechanism of metabolic switch from glucose to lactose was first described in the pioneering work of Jacob and Monod fifty years ago [2]. The operon model that they suggested [3] can be described as follows: In the absence of any regulation, the expression of three structural genes (*lacZ, lacY, lacA*) is inhibited by a repressor molecule, the protein product of *lacI *gene. If present, lactose is taken up from the medium and allolactose, formed from lactose, releases the repressor from the operator. In absence of glucose, cAMP concentration is high and cAMP binds to the catabolite activator protein (CAP), allowing the latter to bind to the promoter and initiate mRNA synthesis. This kind of double control causes the sequential utilization of the two sugars in discrete growth phases. According to this model, the operator region is not essential for operon activity, but rather serves as a controlling site superimposed on a functioning unit [4].

While previous studies were focused on discovery of genetic mechanisms of metabolic switches, we used a new label-free proteomic approach to study the dynamics of protein expression during the metabolic switch. Proteomics is a powerful and rapidly developing field of research, increasingly expanding our detailed understanding of biological systems. It can be used in basic studies on protein dynamics, localization, and function [5] but also to discover potential biomarkers for diseases and response to pharmaceuticals [6]. Proteomics aims to be *comprehensive *- quantifying "all" proteins present in an organism, tissue or cell. This is a non-trivial task, as there are no amplification methods akin to the polymerase chain reaction available, and proteins in a complex sample typically vary over many orders of magnitude in concentration. Common solutions to overcome this problem include fractionation of proteins, *e.g*. by SDS-PAGE [7] or chromatography, and depletion of abundant proteins [8,9]. The accurate quantitation of changes in protein expression in or between different samples or states is one of the primary objectives in proteomics [10]. Several methods for labeling proteins metabolically (in cell cultures) or after extraction are widely applied in "shotgun" proteomics. The labels either incorporate heavy, stable isotopes or a fluorescent group. Nonetheless, it is also possible to quantify peptides and proteins in individual samples directly from the mass spectrometer signal, the so-called "label-free" quantitation. This type of quantitation demands reproducible sample preparation and protein digestion, and benefits from using a mass spectrometer with a wide dynamic range and resolving power, such as an FTICR instrument. Despite these prerequisites, label-free quantitation holds a few advantages over the use of labels. For instance, the sample workup procedure is simpler as there is no labeling step, and the number of samples is not in any way limited by number of labeling reagents and can be used in large studies or for analyzing a large number of time points. Methods based on labeling, on the other hand, have a built-in maximum number of samples that can be analyzed in parallel, beyond which multiple analyses has to be made by bridging between them (which requires one sample or reference to be shared between at least two analyses). Label-free methods seek to reduce potential interferences, for instance by increasing resolving power, and improving accuracy, *e.g*. through data normalization [11]. In our study we used a novel FTICR-ion trap cluster which combines the high mass accuracy of FTICR with fast and relatively inexpensive ion traps for MS/MS [12] making it ideally suited for large-scale, label-free proteomic studies.

## Results and Discussion

**Figure 1:**
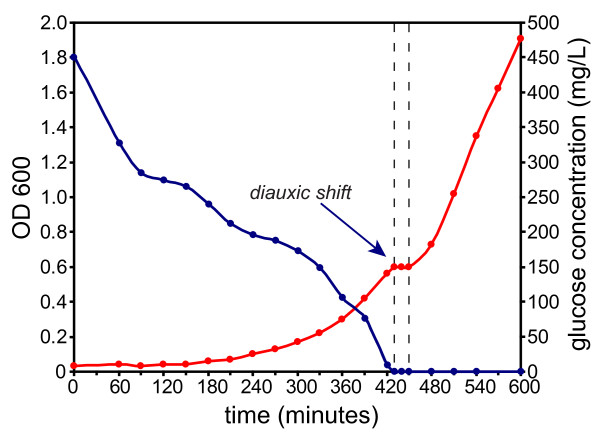
**Measured cell growth and glucose concentration**. Measured cell growth (OD600, blue) and glucose concentration (red) in one glucose-lactose diauxie experiment. The onset of the diauxic shift is easily determined from the 20-30 minute plateau in the growth curve, which coincides with the depletion of glucose in the medium. After about +200 minutes, both sugars are exhausted and the growth stops (OD600_max _= 2.2-2.4).

**Figure 2:**
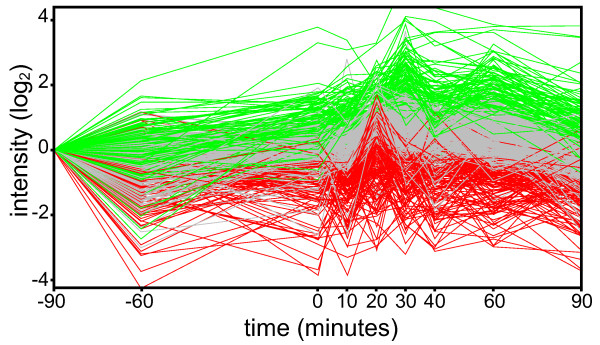
**Glucose-lactose diauxie protein expression**. The proteins expressions were visualized using R and clustered in three groups (green - upregulated, red - downregulated, gray - no change). For subsequent analyses, the time scales of all replicates were aligned with time *t *= 0 at the observed onset of diauxic shift.

**Figure 3:**
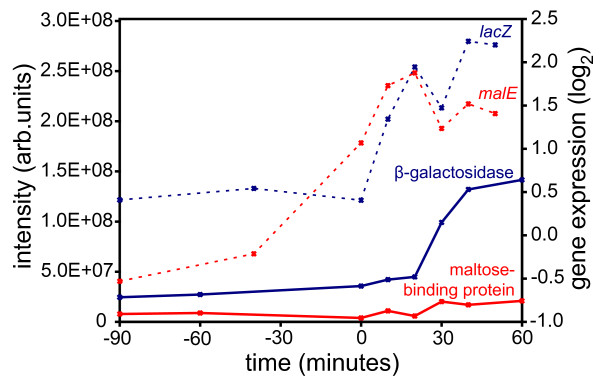
**Expression of *lacZ *and *male***. mRNA (dashed) and protein (solid) dynamics for periplasmic maltose-binding protein/*malE *(red) and β-galactosidase/*lacZ *(blue) upregulated during glucose-lactose diauxie (time 0).

The glucose-lactose diauxie is a classical *Escherichia coli *experiment which has been repeated many times, including recent studies on gene expression using microarrays [13]. In our experimental setup, the growth rate and glucose concentration allowed precise determination of onset of glucose-lactose (Figure 1). The onset of diauxie occurred when cell suspension reached OD600 of ~0.6 or a density of approximately 5 × 10^8 ^cells/mL [14]. This was reproducible in each experiment (OD600 of 0.64, 0.60, and 0.55 respectively) and the OD600 could be used as a predictor during the experiment to optimize the sampling of the culture before and during the diauxic shift. The cell density at the onset of diauxic shift was approximately one quarter of the final density, which is consistent with previous observations, and depends on the glucose-lactose ratio [15]. The time scales for all protein expression measurements could thus be aligned to the growth curve for each replicate culture experiment, facilitating discrimination consistent observations and measurement noise. From the LC-MS/MS data of 52 SDS-PAGE slices, 4,333 peptides from 948 proteins were identified (see the additional file 1) with a false discovery rate of 6.75% of the peptide level (Figure 2). During the diauxie, we observed rapid changes in protein expression (see the additional file 2). However the magnitude of those changes was not as drastic as gene expression. Comparing with the publicly available gene expression data from Traxler *et al*. [13], many similar expression patterns can be recognized, especially for strongly upregulated genes/proteins. Not surprisingly, β-galactosidase expression increased strongly, almost 16-fold, during diauxic shift and followed the dynamics of gene expression (Figure 3) with a small lag expected by the delay between gene activation and accumulated protein. The genetic response occurred immediately after glucose exhaustion but protein synthesis is typically delayed between 20 seconds and several minutes in *E. coli *[3]. Small relative changes in concentration of already abundant proteins are difficult to detect immediately and need to be accumulated for some time before they can be observed. Nevertheless, we noticed that the most significant changes in protein abundance took place within 40 minutes after onset of diauxic shift, which is consistent with published gene expression data and the observed resuming of growth. Since the gene expression data was derived from that published by Traxler *et al*., the alignments of the time-scales are not perfect and minor discrepancies between the sampling of the gene and protein expression could be expected. The protein expression measurements were with a few exceptions reproducible, albeit not always in perfect agreement with the published gene expression data. This could be explained by noise in the data and the fact that gene and protein expression were not measured in the same cell culture. For instance, the change in gene expression of *malE *is almost the same as for *lacZ*, but at the proteomic level we observed only slight changes in abundance of the maltose-binding protein coded for by *malE *(Figure 3). (The maltose-binding protein is a periplasmic component of the maltose ABC transporter which is capable of transporting malto-oligosaccharides up to seven glucose units long [16].)

Using the clustering function for large datasets, *clara*, from the R *cluster *package [17], the dataset could be broadly divided into groups of up- and downregulated proteins, along with proteins that do not change measurably as a function of the diauxic shift. The FTICR-ion trap cluster provided comprehensive label-free quantitative proteomic data with sufficient throughput for an arbitrary number of conditions or time points and biological replicates (here about 30), allowing a global study of protein expression dynamics in *E. coli*. With this instrument platform, proteomics data such as that presented here can be routinely generated in less than 48 h.

**Figure 4:**
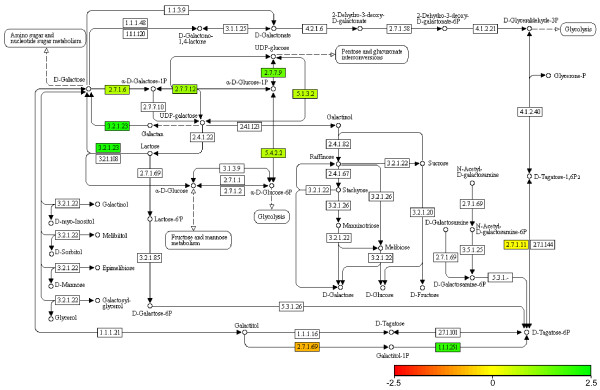
**Protein expression mapped onto KEGG pathway**. The protein expression profiles mapped onto the galactosidase metabolic pathways highlights changes in metabolism when shifting from glucose to lactose as primary carbon source. The measured changes in enzyme (protein) abundance were converted to color and mapped onto KEGG pathways. Upregulated proteins are marked in green, downregulated in red, and unchanged in yellow.

To illustrate changes in metabolic pathways, the protein expression data was mapped onto KEGG metabolic pathways and changes in level of expression indicated by color (Figure 4). Most proteins in the same pathways as β-galactosidase were also markedly upregulated, leading to a global activation of the galactose pathway responsible for channeling lactose into the glycolytic pathway. Other metabolic pathways changed to a lesser degree, as measured by protein (enzyme) abundance.

## Conclusions

We have reproduced the textbook glucose-lactose diauxie experiment in *E. coli *using a state-of-the-art method for quantitative proteomics using a novel mass spectrometry platform, the FTICR-ion trap cluster. In each of three experiments the onset of diauxie occurred at approximately the same cell density and the duration of diauxic shift was also similar. The identified and individually quantified peptides were collected into quantitative protein measurements, which were visualized and compared using tools developed in-house. Through kind assistance from KEGG it is now possible to upload color codes for a whole list of quantified proteins on any metabolic pathway overview (the R program for generating the color codes from protein abundance ratios is available from the authors). We could confirm that the most strongly induced enzymes belong to the pathway responsible for glucose and lactose metabolism.

The FTICR-ion trap cluster in combination with the appropriate visualization tools makes an efficient approach for investigation of protein expression dynamics. The new instrument configuration and software proved robust in acquiring and processing data, allowing label-free quantitation of ~1,000 identified proteins over ~30 time points in a 24 h measurement. Furthermore, the high dynamic range and resolving power of FTICR made label-free quantitation accurate and precise, at least for a label-free method [18]. Finally, as expected, key aspects of the proteome dynamics were indeed bound to reflect gene expression under the glucose-lactose metabolic switch.

## Methods

### *Escherichia coli *Glucose-Lactose Diauxie Experiment

Previous work has shown that glucose-lactose diauxie involves activation of the *lac *operon and high expression of β-galactosidase, but also of many other genes and proteins. To compare with gene expression data we reproduced the experiment of Traxler *et al*. using *E. coli *K12 strain MG1655 (ATCC^® ^Number 47076, ATCC, Manassas, VA, USA); this strain was grown overnight in 25 mL Luria-Bertani (LB) medium in 50-mL Falcon tubes. When optical density at 600 nm (OD600) reached 5.0, the cell culture from each Falcon tube was spun down in an Eppendorf 5810 centrifuge at 194 × g and 37°C. The supernatants were removed, the pellets resuspended in warm (37°C) sterile PBS, pooled together and spun down again with the same parameters. After the PBS was removed, 10 ml of 1× MOPS minimal medium (Teknova, Hollister, CA, USA) was added and the OD600 measured. This culture was then used to inoculate a 3-L bioreactor (Applikon, Schiedam, Netherlands) with 1 L 1× MOPS minimal medium containing 0.5 g/L glucose and 1.5 g/L lactose as the only carbon sources. The temperature was kept at 37°C, dissolved oxygen maintained above 20% and the growth of cells monitored by sampling 1.5 mL of culture for OD600 measurement. The concentration of glucose and lactose were assayed using enzymatic kits (Sigma-Aldrich, St. Louis, MO, USA

and BioVision, Mountain View, CA, USA, respectively). Samples were drawn from the culture every 30 minutes before and after diauxie and every 10 minutes near and during the diauxic shift. Cells were spun down at 4°C and 3,500 rpm, transferred to a fresh tube and frozen at -20°C. After collection of all time points, all pellets were thawed, rinsed with ice cold PBS, transferred to a 1.5-mL Eppendorf tube and spun down again for 10 min on maximum speed (16,100 × g) at 4°C.

### Protein Extraction, In-solution and In-gel Digestion

The pellets were weighed and 5 mL of the BugBuster^® ^Master Mix (Novagen, Merck KGaA, Germany) was added per gram cell paste. Cells were incubated at room temperature on a shaking platform at slow settings for 20 min. After the insoluble cell debris was removed by centrifugation at 16,100 × *g *for 20 min at 4°C, the supernatant was transferred to a fresh tube. Proteins extracted from the pooled sample of one early and one late time point were used for SDS-PAGE protein separation and in-gel digestion for peptide and protein identification. The rest of the proteins were used for in-solution digestion and peptide and protein quantitation. The extracted proteins for each time point and replicate were digested using trypsin. To each 50 μL of protein extract (approximately 0.25 mg protein) 10 μL 60 mM DTT in 25 mM ammonium bicarbonate (ABC) was added, followed by incubation for 45 min at 56°C to reduce cystines. After 45 minutes, 100 mM iodoacetamide (IAA) in ABC was added to a final IAA concentration 25 mM and the samples kept in dark for 1 h at room temperature to alkylate and protect the cysteins. The proteins were then digested for 5 hours at 37°C by adding 10 μL 100 ng/μL sequencing-grade trypsin (sequencing grade, Promega, Madison, WI, USA) in ABC. The digestion was quenched by adding 5 μL 10% TFA to lower the pH. The peptide digests were stored at -20°C until analysis.

For MS/MS peptide identification, 25 μg of proteins from two time points, one before and one after the diauxic shift, were fractionated using 8-12% acrylamide SDS-PAGE (NuPAGE™ 8-12%, Invitrogen, Carlsbad, CA, USA). The gel was stained overnight (12 h) in staining solution (Invitrogen) with 5% methanol and was then washed with milli-Q water until cleared. The gel lanes were cut into twenty-six 2 mm bands and transferred to 96-well plate. Each band was de-stained using 25 mM ABC and acetonitrile, reduced (75 μL 10 mM DTT, 56°C, 30 minutes), alkylated (75 μL 55 mM iodoacetamide, room temperature, 20 min in dark) and digested in-gel using trypsin (20 μg in 20 μL) 12 h at 37°C. The supernatant from each well was transferred to a fresh plate. The digestions were quenched by adding 4 μL 5% TFA (first extraction). The gel pieces were then incubated for 1 hour at 37°C in 0.1% TFA, after which the second supernatant was pooled with the first extraction and frozen.

### FTICR - Ion Trap Cluster

The novel FTICR - ion trap cluster [12] consists of a refrigerated solariX™ 12 T FTICR (Bruker Daltonics, Bremen, Germany) and six ion traps. In this study, CID data from an HCT ultra ion trap (Bruker Daltonics) was used for peptide identification by MS/MS. All mass spectrometers in the cluster were coupled on-line to parallel, splitless NanoLC-Ultra 2D plus systems (Eksigent, Dublin, CA, USA) with additional loading pumps for fast sample loading and washing, which resulted efficient use of the mass spectrometers and high chromatographic peak capacity. All LC systems were configured with 15-cm 300 μm-i.d. ChromXP C18 columns supplied by Eksigent and linear 90 minute gradients from 4 to 44% acetonitrile in 0.05% formic acid were applied. The LC systems were controlled by HyStar 3.2-3.4 with a plugin from the LC manufacturer, the ion traps by esquireControl 6.2 and the FTICR by apexControl 3.0, all from Bruker. The acquired data from each mass spectrometer was automatically transferred to a dedicated server and processed as described below.

### Data analysis

**Figure 5:**
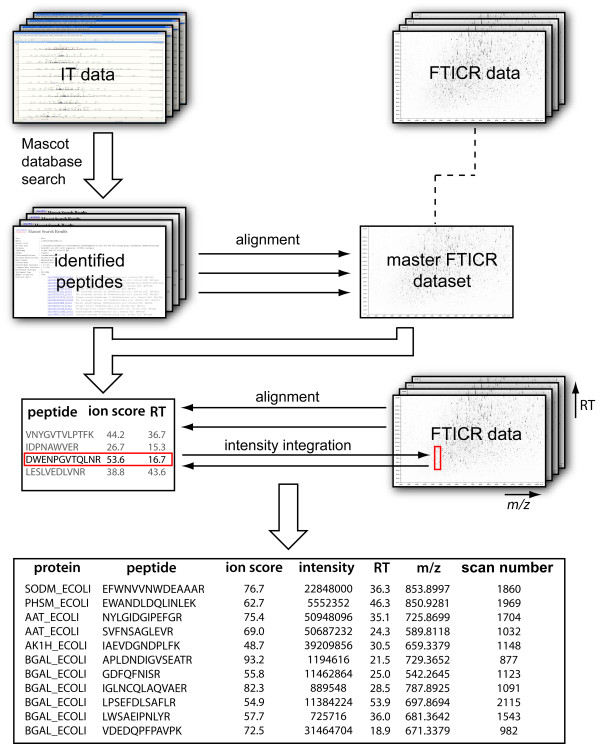
**Data processing workflow**. The data obtained from the FTICR-ion trap cluster was processed using the workflow illustrated here. First, the LC-MS/MS datasets from the ion trap were searched against the *Escherichia coli *protein sequence database using Mascot. Each individual result was aligned to a single master LC-MS dataset and then merged into one file with aligned retention times. Each separate FTICR LC-MS dataset was aligned against the merged LC-MS/MS data (and hence the master FTICR dataset). Intensities of the identified peptides were then extracted from each FTICR LC-MS dataset by taking the maximum signal in a window of defined *m/z *and retention time relative to the identified peptide. The resulting list contained the protein name, peptide sequence, maximum observed ion score, and absolute intensities for each peptide. This information from each sample could then easily be collapsed into a single, uniform sample/data matrix with the total absolute intensities for all identified proteins and samples.

Each individual MS/MS dataset provided by the ion traps was converted to MGF files using DataAnalysis (Bruker Daltonics). The datasets were separately searched using Mascot 2.1 and converted to the pepXML [19] format. Using the identified peptides, each LC-MS/MS dataset was aligned against a master FTICR LC-MS dataset using msalign [20] and merged. All identified peptides with a best Mascot ion score of at least 25 were then aligned against each individual FTICR LC-MS dataset, one for each biological replicate and time point. Using these alignments, the peaks corresponding to the identified peptides were integrated over the duration of the chromatographic peak. The data analysis workflow is illustrated in Figure 5. Only peptide identifications confirmed by accurate mass measurement were thus used. The peptides were then grouped into proteins, using only peptides attributable to a single protein, and the sum of all peptide intensities used as a measure of protein abundance. The data was normalized against the most abundant protein and the earliest time point. The resulting relative protein intensities were log_2_-transformed and visualized using the *gplots *package in R. In the same package we created hexadecimal color codes corresponding to the average values over all expression ratios for each protein. An expression ratio of +2.5 thus corresponded to #00FF00, 0 to #FFFF00 and -2.5 to #FF0000. The color codes were then mapped onto metabolic pathways available in the Kyoto Encyclopedia of Genes and Genomes (KEGG) [21].

## Authors' contributions

EM carried out sample preparation, data acquisition, analysis and interpretation, and drafted the manuscript. MP conceived of the study, and participated in its design and coordination, carried out data analysis and helped draft the manuscript. AD supervised the work and critically revised the manuscript. All authors read and approved the final manuscript.

## Supplementary Material

Additional file 1 **Peptides identifications**. The file represents peptide identifications obtained after Mascot search of all IT LC-MS/MS data and alignment to master FTICR LC-MS dataset. Click here for file

Additional file 2 **Summarized peak intensities**. The file provides absolute intensities for a list of all identified proteins in each experiment at each time point. Click here for file
